# Correction: Chondrogenesis of Infrapatellar Fat Pad Derived Adipose Stem Cells in 3D Printed Chitosan Scaffold

**DOI:** 10.1371/journal.pone.0102638

**Published:** 2014-07-07

**Authors:** The PLOS ONE Staff

## Notice of Republication

This article was republished on June 18, 2014, due to Figure 6 and Figure 7 being ordered incorrectly. Please download the PDF again to view the corrected article. The originally published, uncorrected article and the republished, corrected article are provided here for reference.

## Supporting Information

File S1 Originally published, uncorrected article
(PDF)

File S2 Republished, corrected article
(PDF)
