# Supplementary material for: Correction: Chondrogenesis of Infrapatellar Fat Pad Derived Adipose Stem Cells in 3D Printed Chitosan Scaffold
Source: PLoS One. 2014 Jul 7;9(7):e102638. doi: 10.1371/journal.pone.0102638 (PMC4085024; doi:10.1371/journal.pone.0102638)
Supplement: File S2 — Republished, corrected article (PDF) [file pone.0102638.s002.pdf]

OPEN ACCESS Freely available online

PLOS | ONE

Chondrogenesis of Infrapatellar Fat Pad Derived Adipose Stem Cells in 3D Printed Chitosan Scaffold

Ken Ye[1,2], Raed Felimban[1,2], Kathy Traianedes[3], Simon E. Moulton[4], Gordon G. Wallace[4], Johnson Chung[4], Anita Quigley[3], Peter F. M. Choong[1,2], Damian E. Myers[1,2]*

1 Department of Surgery, St Vincent's Hospital, University of Melbourne, Fitzroy, Victoria, Australia, **2** Department of Orthopaedics, St Vincent's Hospital, Fitzroy, Victoria, Australia, **3** Departments of Medicine and Clinical Neurosciences, St Vincent's Hospital, University of Melbourne, Fitzroy, Victoria, Australia, **4** Intelligent Polymer Research Institute, University of Wollongong, ARC Centre of Excellence for Electromaterials Science (ACES), Squires Way, North Wollongong, New South Wales, Australia

Abstract

Infrapatellar fat pad adipose stem cells (IPFP-ASCs) have been shown to harbor chondrogenic potential. When combined with 3D polymeric structures, the stem cells provide a source of stem cells to engineer 3D tissues for cartilage repair. In this study, we have shown human IPFP-ASCs seeded onto 3D printed chitosan scaffolds can undergo chondrogenesis using TGFβ3 and BMP6. By week 4, a pearlescent, cartilage-like matrix had formed that penetrated the top layers of the chitosan scaffold forming a 'cap' on the scaffold. Chondrocytic morphology showed typical cells encased in extracellular matrix which stained positively with toluidine blue. Immunohistochemistry demonstrated positive staining for collagen type II and cartilage proteoglycans, as well as collagen type I. Real time PCR analysis showed up-regulation of collagen type II, aggrecan and SOX9 genes when IPFP-ASCs were stimulated by TGFβ3 and BMP6. Thus, IPFP-ASCs can successfully undergo chondrogenesis using TGFβ3 and BMP6 and the cartilage-like tissue that forms on the surface of 3D-printed chitosan scaffold may prove useful as an osteochondral graft.

Citation: Ye K, Felimban R, Traianedes K, Moulton SE, Wallace GG, et al. (2014) Chondrogenesis of Infrapatellar Fat Pad Derived Adipose Stem Cells in 3D Printed Chitosan Scaffold. PLoS ONE 9(6): e99410. doi:10.1371/journal.pone.0099410

Editor: Gwendolen Reilly, University of Sheffield, United Kingdom

Received February 20, 2014; **Accepted** May 14, 2014; **Published** June 11, 2014

Copyright: © 2014 Ye et al. This is an open-access article distributed under the terms of the Creative Commons Attribution License, which permits unrestricted use, distribution, and reproduction in any medium, provided the original author and source are credited.

Funding: This work was funded through the Australian Orthopaedics Association Research Foundation (AOA Research Foundation), Australian Research Council (ARC) and National Health and Medical Research Council (NHMRC) Postgraduate Scholarship for author K Ye (APP1017633). The authors have no other relevant affiliations or financial involvement with any organization or entity with a financial interest in or financial conflict with the subject matter or materials discussed in the manuscript apart from those disclosed. No writing assistance was utilized in the production of this manuscript. The funders had no role in study design, data collection and analysis, decision to publish, or preparation of the manuscript.

Competing Interests: The authors have declared that no competing interests exist.

* Email: damianem@unimelb.edu.au

Introduction

Articular cartilage defects have limited capacity for self-regeneration and healing. Cartilage damage often results in pain and loss of function for the patient and often accelerates the development of osteoarthritis in the joint. Current methods of osteochondral repair aimed at improving symptoms and function include microfracture, osteochondral grafting, and autologous chondrocyte transplantation (ACT) [1,2,3,4,5,6]. However, there are inadequacies with these procedures which include the formation of fibrocartilage, donor site morbidity, hypertrophy of implant, and suboptimal long term outcomes [7,8,9,10,11].

Tissue engineering may offer treatment options that could overcome the limitations of current management options. The combination of cells, scaffold and biochemical factors may provide the possibility of true cartilage regeneration. Although the use of autologous chondrocytes has yielded some good short term results, long term results are equivocal [12,13,14,15,16]. Furthermore the use of autologous chondrocytes is limited by major factors, including donor site morbidity, and chondrocytes are limited in number comprising of only 5–10% of cartilage tissue, thus require expansion which may lead to dedifferentiation [7,17,18,19,20]. Due to these limitations, mature chondrocytes are not ideal candidate cells to use in tissue engineering constructs.

Adult mesenchymal stem cells can overcome some of the aforementioned issues. These cells can be derived from bone marrow, fat, skin, muscle, periosteum, or cord blood [21,22,23,24,25,26]. More recently, adipose tissue has become an attractive source of adipose stem cells (ASC) due to the ease of accessibility and great abundance [27,28]. Compared to bone marrow, adipose tissue is reported to give a higher yield of stem cells [29]. These cells have enormous capacity for proliferation and differentiation into chondrocytes as shown in many groups [27,28,30] Most have used ASCs derived from the stromal vascular fraction (SVF) of liposuction [31]. However, some have used adipose stem cells derived from the infrapatellar fat pad (IPFP) during total knee arthroplasty because the removal of IPFP improves surgical access and visualization, and reduces the chance of impingement of the fat pad by the prosthesis. It may be that this autologous source of stem cells is a suitable candidate cell for repairing cartilage defects in the knee before total knee arthroplasty is required and may form part of a one-step surgical procedure for autologous stem cell transplantation in the knee [32].

Chitosan has been used widely in the tissue engineering field for cartilage engineering [33]. It shares some structural characteristics with various glycosaminoglycans and hyaluronic acid found in native cartilage. It is usually biocompatible and degradation products are often elements involved in the synthesis of cartilage,

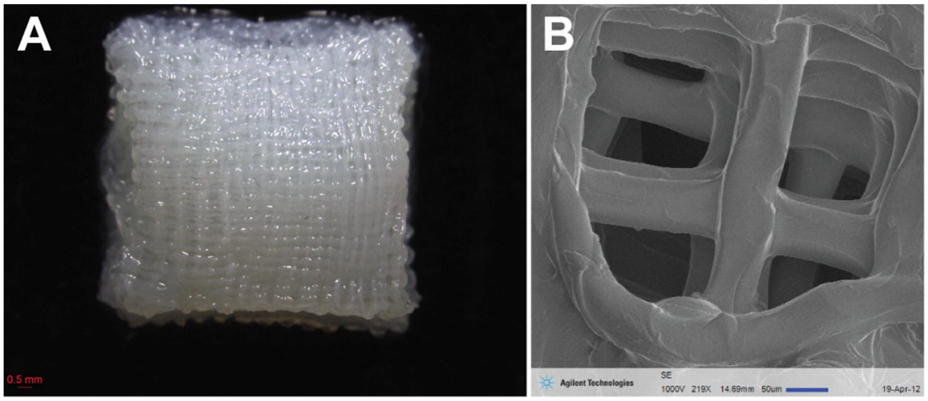

Figure 1. 3D printed chitosan scaffold. (A) Macroscopic image of a 3D printed chitosan scaffold showing strands of chitosan extruded in a 3D lattice pattern overlying each other forming a 3D structure. (B) Scanning electron microscope (SEM) image showing the lattice network of chitosan fibres. Scale bars as indicated.
doi:10.1371/journal.pone.0099410.g001

such as chondroitin sulfate, hyaluronic acid, keratin sulfate and glycosylated collagne type II) [34]. 3D structures can be designed to mimic the native cartilage environment and thus, in theory, should provide greater chance for cartilage regeneration [35]. Some studies have shown that chondrocytes require a 3D environment to avoid dedifferentiation [20]. 3D printed structures can also be engineered using computer-assisted drawing technologies (AutoCAD) and thus made to any shape or size to fill defects; this greatly enhances their potential clinical use.

In the current study we have used ASC derived from IPFP that was removed during total knee arthroplasty for osteoarthritis. Our aim was to investigate *in vitro* chondrogenesis of IPFP-ASCs using a 3D chitosan engineered scaffold.

Materials and Methods

Ethics Statement

Infrapatellar fat pads were obtained intraoperatively from total knee arthroplasties after informed written consent and approval from Human Research Ethics Committee at St Vincent's Hospital (HREC-A 117/10). All necessary ethics protocols were adhered to in the process of tissue harvest and use. Only patients with primary osteoarthritis were selected. Patients with inflammatory arthritis and with a history of prior knee surgery were excluded from selection. A total of three patients (2 female, 1 male) with mean age of 69 (aged 67, 69, 71; N = 3) were included in this study.

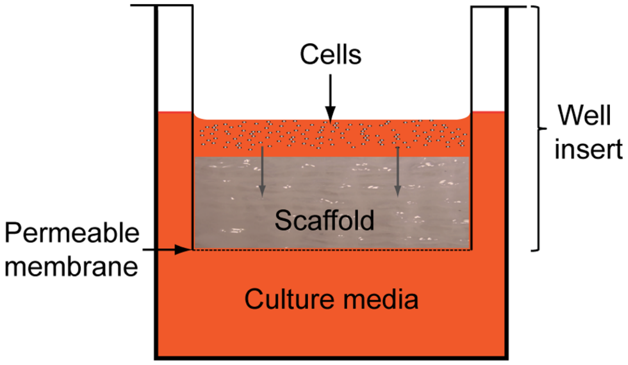

Figure 2. In vitro culture of cell-scaffold constructs using cell inserts. Scaffolds were cut using a 6 mm biopsy punch and seeded with 7.5×10^5 ASCs in a 24 tissue culture plate well inserts with an internal diameter of 6.5 mm and permeable membrane of 3.0 µm pore size.
doi:10.1371/journal.pone.0099410.g002

Materials

Materials used for IPFP-ASC isolation, culture and differentiation are listed as the following: Dulbecco's phosphate-buffered saline (D-PBS), fetal bovine serum (FBS), antibiotic/antimycotic solution (Amphotericin B, Penicillin, Streptomycin 100×), glutamax, L-ascorbic acid 2-phosphate, transforming growth factor beta-3 (TGFβ3), and HEPES buffer were purchased from GIBCO, Life Technologies Corporation (Carlsbad, CA, USA); Red cell lysis buffer, Dulbecco's modified eagle medium (DMEM), insulin-transferring-selenium (ITS), dexamethasone, and 0.1% EDTA/0.25% trypsin were from Sigma-Aldrich (St. Louis, MO, USA). All culture plates, conical tubes, well inserts were from Corning Inc., (NY, USA) and cell filters from Millipore (Darmstadt, Germany). Collagenase type 1 was purchased from Worthington Biochemical Corporation (Lakewood, NJ, USA). Human epidermal growth factor (hEGF), human fibroblastic growth factor-2 (hFGF-2), and bone morphogenetic protein-6 (BMP6) were purchased from R&D Systems, Inc. (Minneapolis, MN, USA).

Histology and immunohistochemistry reagents were purchased as listed: Neutral buffered formalin (NBF), Mayer's haematoxylin and eosin (H&E), toluidine blue were from Sigma-Aldrich (St. Louis, MO, USA). Hydrogen peroxide was from Merck Millipore (Darmstadt, Germany); Proteinase K, rabbit serum, secondary antibodies (biotinylated rabbit polyclonal anti-goat and anti-mouse antibodies), and liquid DAB+ were purchased from Dako (Glostrup, Denmark); Horseradish peroxidase (HRP)-conjugated streptavidin was from the Vectastain ABC kit from Vector Laboratories (Burlingame, CA, USA); Primary antibodies included mouse monoclonal anti-human type II collagen IgG antibody (MP Biomedical, Solon, OH, USA), goat polyclonal anti-human type I collagen IgG antibody (SouthernBiotech, Birmingham, AL, USA), and mouse monoclonal anti-human cartilage proteoglycan IgG antibody (Merck Millipore, Billerica, MA, USA). Goat IgG isotype control was from SouthernBiotech (Birmingham, AL, USA) and mouse IgG isotype control was from Invitrogen, Life Technologies Corporation (Carlsbad, CA, USA).

Reagents for qPCR included Trizol (Ambion, Life Technologies, Carlsbad, CA, USA), and all other RNA extraction materials from Qiagen (Hilden, Germany). cDNA synthesis materials were

from Promega (Madison, WI, USA). Taqman probes were used for the evaluation of collagen type I, II, SOX9, Aggrecan and GAPDH genes (Invitrogen, Life Technologies Corporation, Carlsbad, CA, USA).

Cell Isolation and Culture

Cell isolation and culture was based on a previously published protocol of isolating cells from lipoaspirate material and adapted for the isolation of cells from the IPFP [36]. Briefly, the IPFP was immediately placed in sterile normal saline and processed within 30 minutes of harvest. Initially the tissue was washed several times with PBS, to remove contaminating blood. Fibrous material such as capsule or meniscus were dissected and discarded. The remaining fat content was diced using a scalpel and digested with 0.2% Collagenase Type 1 for three hours at 37C under constant agitation. The released cells, IPFP-ASCs, and materials were filtered through a 100 µm nylon mesh and centrifuged at 400 g at room temperature for five minutes to separate the stromal vascular fraction (SVF) from the floating adipocytes. The supernatant was discarded and the cell pellet resuspended in Red Cell Lysis Buffer and incubated at room temperature for 10 minutes. This was then filtered through a 40 µm nylon mesh before centrifugation at 400 g at room temperature for five minutes. The cells were resuspended in PBS, counted and plated in monolayer culture (75 cm^2 tissue culture flask) at 5 000 cells/cm^2 in stromal media (SM) containing DMEM supplemented with 10% FBS, 1× antibiotic/antimycotic solution, 1× Glutamax, and 15 mM HEPES. Cultures were maintained 48 hours at 37C in 5% CO_2. The cells were washed and media were replaced with expansion media containing stromal media with 5 ng/ml human epidermal growth factor (hEGF) and 1 ng/ml human fibroblastic growth factor (hFGF). The cells were cultured until they reached 80% confluence and then harvested with 0.1% EDTA/0.25% trypsin and made into a single cell suspension for seeding onto the chitosan scaffold.

Scaffold Preparation

A 3% w/v medium molecular weight chitosan solution was prepared in 2% v/v acetic acid (Sigma-Aldrich, St. Louis, MO, USA). The solution was filtered and centrifuged to remove air bubbles before loading into a disposable syringe (Nordson EFD) fitted with a 200 µm diameter nozzle. The chitosan solution was extrusion printed onto a glass slide immersed in a precipitating bath of isopropyl alcohol using a custom modified computer numerical control (CNC) milling machine (Sherline Products, CA). The system was equipped with a three-axis positioning platform and designed using EMC2 software (LinuxCNC). An attachment for syringe deposition was built and connected to a controllable gas flow regulator (1–100 psi). The regulator was controlled using a Pololu SciLabs USB-to-serial microcontroller and with an in-house software interface. Solutions were extrusion printed at approximately 13 Psi onto a glass slide at a feed rate of 150 mm/min, strand spacing of 0.25 mm, to a final size of 10 mm×10 mm×5 mm with a porosity of 250 µm. Scanning electron microscopy was used to image the scaffold to show micro-architecture of the 3D lattice structure using the Agilent 8500 FE-SEM system (Agilent Technologies Inc, Santa Clara, CA, USA) (Figure 1). The extruded 3D scaffolds were then neutralised in a dilution series of ethanol and PBS over a period of two days.

24 scaffolds were made for this experiment. Three to four 6 mm plugs were cut using a skin biopsy punch and randomly allocated. Six scaffolds were used for each time point and for each condition. Two were used for histological analysis. Four were used to harvest RNA for PCR gene analysis. A total of 72 6 mm plugs of scaffolds were used for 3 biological replicates of IPFP ASCs.

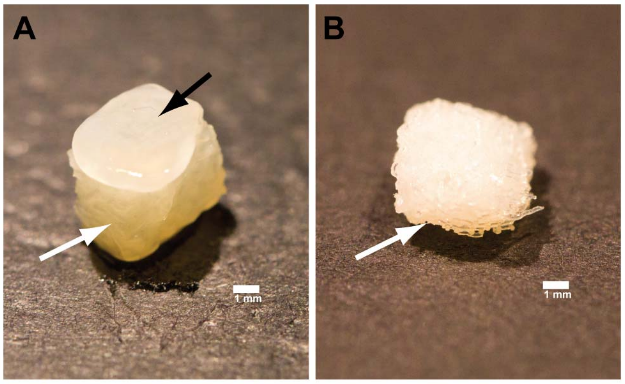

Figure 3. Macroscopic image of cell-scaffold construct at week 4. (A) Macroscopic image showing a cartilage-like cap (black arrow) derived after 7.5×10^5 IPFP-ASCs were seeded onto a 6 mm plug of 3D printed chitosan (white arrow) scaffold to form a cell-scaffold unit in chondrogenic media after 4 weeks. (B) Macroscopic image showing no visible signs of cartilage formation on the chitosan scaffold (white arrow) after 4 weeks in control media without growth factors. The scaffolds were cut using a 6 mm skin biopsy punch. The image was taken using a Canon EOS 55D with macro zoom lens at 100 mm. The scale bar represents 1 mm.
doi:10.1371/journal.pone.0099410.g003

Chondrogenic Differentiation and Culture

Confluent IPFP-ASCs, cells at third passage, were harvested, counted and resuspended in chondrogenic medium (CM) consisting of DMEM-high glucose, 1% FBS, 1% ITS, 100 nM Dexamethasone, 50 µg/ml ascorbic acid, 1× antibiotic/antimy-

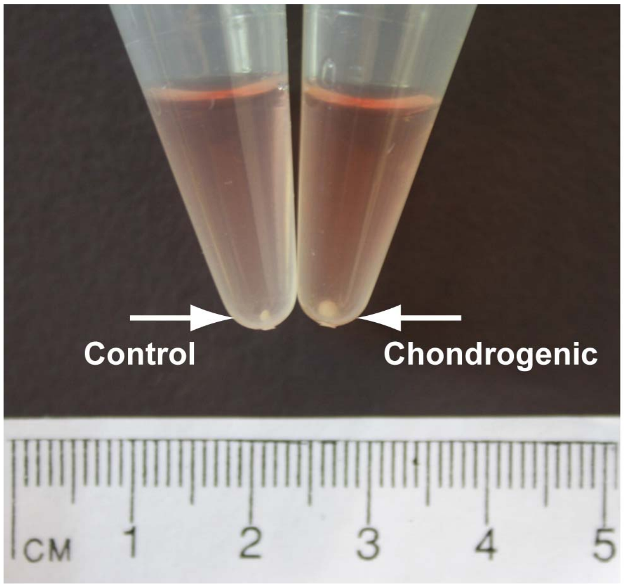

Figure 4. Macroscopic image of pellet cultures in control and chondrogenic media at 4 weeks. A comparative image showing pellets cultured in control vs chondrogenic media shows a distinct difference in size at 4 weeks between the larger chondrogenic pellets and the smaller control pellet.
doi:10.1371/journal.pone.0099410.g004

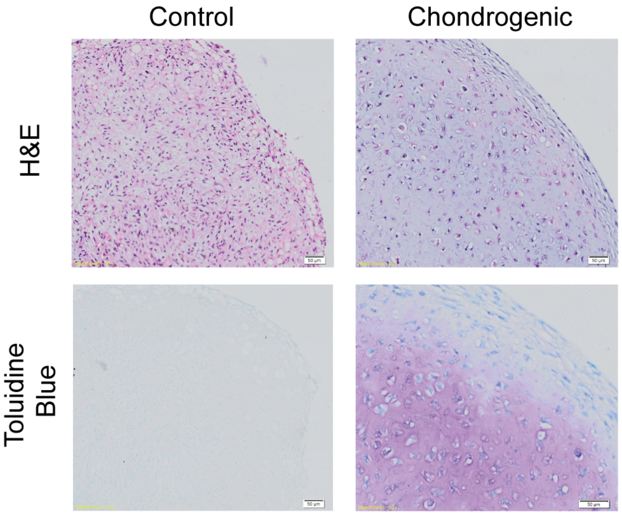

Figure 5. Histology of week 4 pellet culture in control and chondrogenic media. Control media shows cells that appear more fibroblastic in morphology as compared to chondrocytic morphology with extracellular matrix in the chondrogenic pellet. Chondrogenic pellets are larger, well rounded and stains positively for toluidine blue. Magnification 20×. Scale bars at 50 μm as indicated.
doi:10.1371/journal.pone.0099410.g005

cotic, 10 ng/ml TGFβ3 and 10 ng/ml BMP6. Scaffolds were cut using a 6 mm biopsy punch (Kai Medical, Honolulu, HI, USA) and seeded with 7.5×10^5 ASCs in a 24 Transwell tissue culture plate well inserts with an internal diameter of 6.5 mm and 3.0 μm pore size (Figure 2). Cells were seeded on top of the scaffold and 1.2 ml of chondrogenic or control media was used in each well. These were incubated at 37C in 5% CO_2 for 14 and 28 days and media changed three times per week. The same protocol was used for media without growth factors, and served as the negative control. Cells were also cultured by themselves using micromass pellet culture. 250,000 cells were centrifuged at 400 g for 5 minutes in 15 ml centrifuge tubes to form cell pellets and cultured in 0.5 ml of chondrogenic or control media for the same time period as cells cultured on the chitosan scaffolds.

Histology and Immunohistochemistry

After 14 and 28 days of culture, cell pellets and cell-scaffold constructs were harvested for histological and immunohistochemical analysis using standard techniques of fixation, dehydration and paraffin embedding. Pellets and cell-scaffold constructs were fixed in 10% NBF overnight at 4C and processed and imbedded at the histopathology laboratory, pathology department at St Vincent's Hospital, Melbourne, sectioned into 6 μm sections and incubated overnight at 37C. The sections were deparaffinised, rehydrated through graded ethanol, and stained with haematoxylin & eosin (H&E) and toluidine blue (TB) (for glycosaminoglycans (GAGs)).

Accumulation of collagen types I and II, and cartilage-specific proteoglycan was assessed by immunohistochemistry. Sections were treated with 0.3% hydrogen peroxide (H_2O_2) for five minutes, antigen retrieval using Proteinase K for four minutes and were blocked using 10% normal rabbit serum (NRS) for 30 minutes at room temperature. These sections were incubated with the following primary antibodies; mouse monoclonal anti-human type II collagen antibody (1:500), goat polyclonal anti-human type I collagen (1:500), and mouse monoclonal anti-human cartilage proteoglycan antibody (1:500) for 60 minutes at 37C. Isotype negative controls were used at the same concentration as their respective primary antibodies. Subsequently, sections were incubated using biotinylated rabbit polyclonal anti- goat and rabbit anti-mouse antibodies as secondary antibodies for 30 minutes followed by horseradish peroxidase (HRP)-conjugated streptavidin treatment according to the manufacturer's instructions. The reaction was developed as a brown precipitation using peroxidase substrate 3,3-diaminobenzidine (DAB) for 5 minutes. Sections were counterstained with haematoxylin, dehydrated, cleared, and mounted with Pertex.

Quantitative Real Time PCR (qPCR)

Week 2 and 4 cell-scaffold constructs were pulverized in liquid nitrogen using a small mortar and pestle and then homogenized in 1 ml of Trizol solution, and RNA was extracted and purified using a combination of the Trizol method and silica membrane-based commercial extraction kit (QIAGEN, RNeasy mini kit) according to the manufacturer's protocol. RNA from pre-differentiated cells (day 0) was also extracted as Time zero samples. The RNA concentration and purity were measured using a NanoDrop spectrophotometer (Peqlab, Erlangen, Germany) and the Agilent 2100 BioAnalyzer (Agilent Technologies, Santa Clara, CA, USA). Complimentary DNA copies were reverse transcribed from

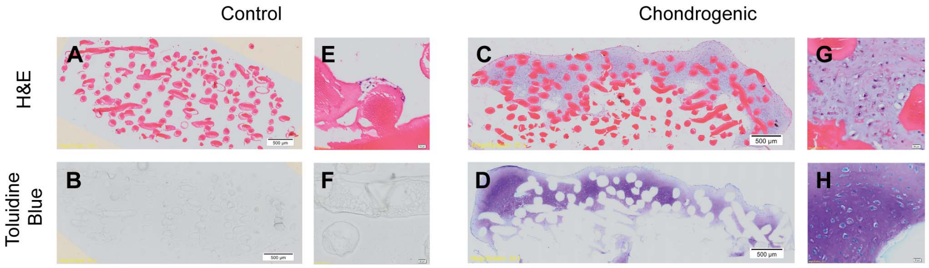

Figure 6. Histology of week 4 chitosan-ASC constructs in chondrogenic and control media. There is a paucity of cellular attachment and growth in control media and lack of toluidine blue staining. Chitosan fibres are clearly stained by eosin. The cells that have grown and attached to the chitosan structure under chondrogenic media shows extracellular matrix deposition with strong toluidine blue staining, suggesting the presence of proteoglycans. Magnification 20×. A–D represent whole tissue images with scale bars at 500 μm as indicated. E–F represents magnified images with scale bars at 20 μm as indicated.
doi:10.1371/journal.pone.0099410.g006

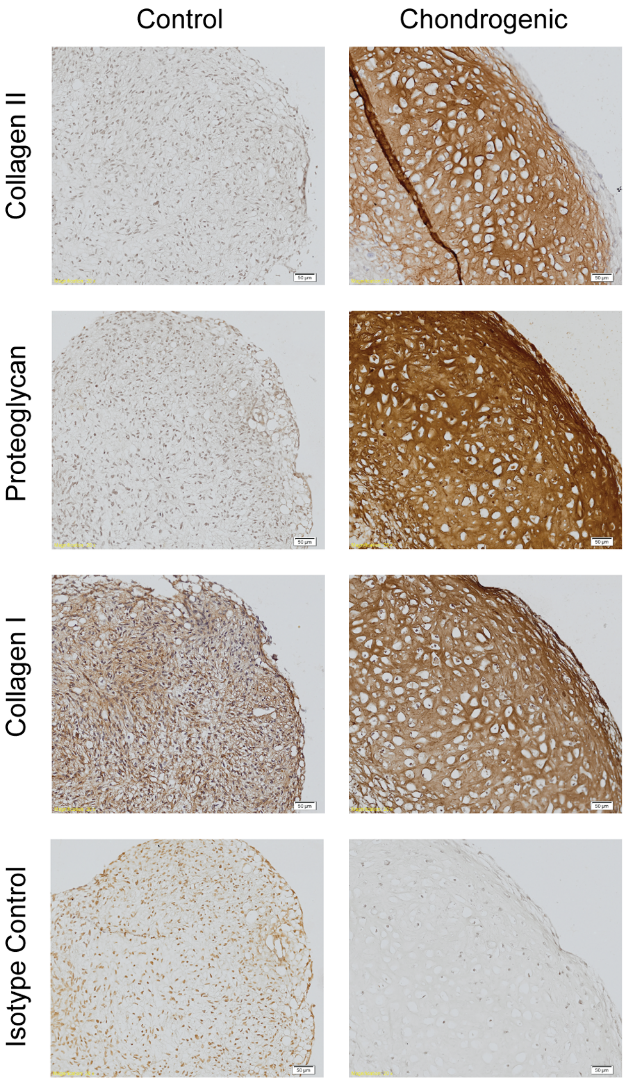

Figure 7. Immunohistochemistry of week 4 pellet culture in control and chondrogenic media. Collagen type II and proteoglycan is expressed in chondrogenic pellet compared with no expression in control media. Collagen type I is expressed in both control and chondrogenic pellet. Magnification 20×. Scale bars at 50 μm as indicated.
doi:10.1371/journal.pone.0099410.g007

200 ng total RNA for all samples using oligo-dT primers and omniscript reverse transcriptase kit according to the recommendations of the manufacturer. qPCR was performed using standard TaqMan Probe-Based Gene Expression Analysis protocols using commercial available probes for collagen types I and II, SOX 9, and Aggrecan. The Taqman primer ID for each gene was as follows: COL1A2 (Hs00164099_m1), COL2A1 (Hs00264051_m1), SOX9 (Hs01165814_m1), and ACAN (Hs00153936_m1). GAPDH was used as housekeeping gene for relative quantification of gene expression (Hs02758991_g1). Liquid handling was performed by the CAS1200 series robot by Corbett Robotics (Corbett Life Sciences, Qiagen, Hilden, Germany). Subsequent PCR reaction was performed using the Lightcycler 480 (Roche, Basel, Switzerland).

Data Analysis

Relative quantification was derived and analyzed using the Second Derivative Maximum method through the Lightcycler 480 software version 1.5. Subsequent numerical data analysis of relative quantification of qPCR results was performed in Microsoft Excel 2010 and GraphPad Prism 6.0 (GraphPad Software, La Jolla, CA, USA) using the $2^{(-\Delta CT)}$ method. Means, standard deviations (SD) and errors (SEM), and 95% confidence limits were calculated for each set of results. The t-test was used to assess significant between two sets of data. Friedman's test was used to assess significance over three or more sets of data. Statistical testing was verified by a statistician.

Results

Cell Culture

IPFP-ASCs cultured for up to four weeks on a 3D chitosan scaffold in chondrogenic media (TGFβ3 and BMP6), developed a pearlescent, white and shiny cartilage-like tissue 'cap' (Figure 3A). Conversely, cell-scaffold constructs cultured in control media showed no visible signs of cartilage-like tissue formation (Figure 3B). Similarly, chondrogenic pellets were larger and rounder than control pellets and also had a shiny white appearance macroscopically (Figure 4).

Histology and Immunohistochemistry

H&E staining of chondrogenic pellets showed a change in cellular morphology towards a chondrocytic phenotype showing larger cells encapsulated in lacunae when compared to control pellets. Positive toluidine blue of chondrogenic pellets contrasts with a lack of toluidine blue staining in control pellets. Furthermore, in chondrogenic pellets, gradient differentiation of toluidine blue staining intensity can be seen on magnification, together with more tangential morphology of cells near the surface (Figure 5).

H&E staining of the cell-scaffold construct show a paucity of cells attached to the chitosan in the negative control, and the cells that did attach appeared to be fibroblastic in appearance and lacked features of chondrocytic morphology. In contrast, cell-scaffold constructs cultured in chondrogenic media showed cellular aggregation throughout the upper layers of the chitosan scaffold forming what can be seen macroscopically as a 'cap' of cartilaginous tissue. These cells exhibit features of chondrocytic morphology as seen in the chondrogenic pellets as well as staining for toluidine blue (Figure 6). The chitosan fibres were stained pink by the eosin and therefore easily recognizable in the H&E stain. The chitosan fibres did not stain for toluidine blue and therefore appeared like empty spaces in the toluidine blue stain.

Immunohistochemistry staining of chondrogenic and control pellets for the presence of collagen type II, cartilage proteoglycan, and collagen type I at week 4 is shown in Figure 7. When compared to control pellets and isotype controls, chondrogenic pellets stained strongly for collagen type II and proteoglycan in the extracellular matrix. Collagen type I was present in both the control and the chondrogenic pellets. Similar patterns of staining can be seen in the cell-scaffold constructs (Figure 8). Strong staining for collagen type II and cartilage proteoglycans is seen throughout cartilaginous 'cap'. There was also staining of collagen type I throughout, which appears to co-localise with collagen type II in some parts. All immunohistochemistry staining show the cells are encased in their lacunae consisting of extracellular matrix consisting of predominantly collagen type II. Some non-specific staining of chitosan fibres is also present in the proteoglycan stain.

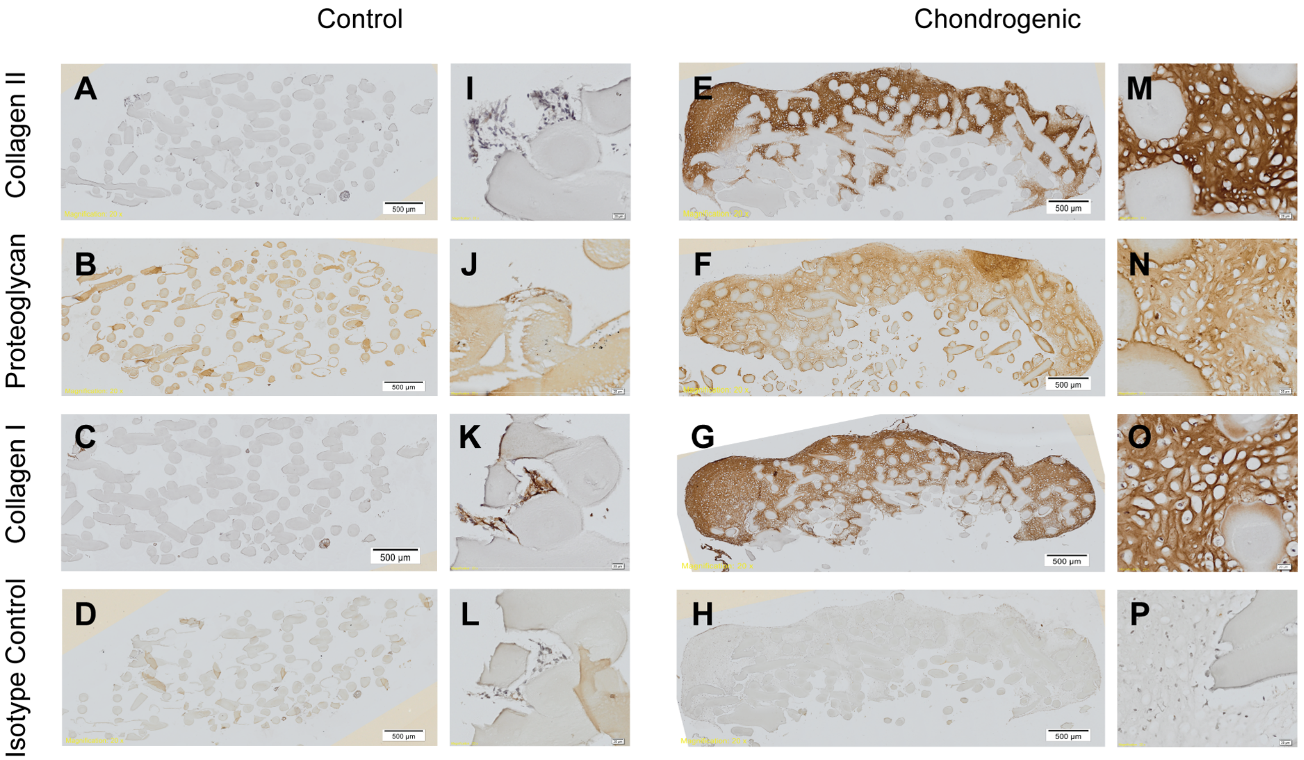

Figure 8. Immunohistochemistry of week 4 chitosan-ASC constructs in chondrogenic and control media. There is a paucity of cellular attachment and growth in control media and lack of collagen type II and proteoglycan expression. The cells that have grown and attached to the chitosan structure under chondrogenic media strongly expresses collagen type II, proteoglycan and also collagen type I, indicating the formation of hyaline-like cartilage. Magnification 20×. A-H represent whole tissue images with scale bars at 500 µm as indicated. I-P represent magnified images with scale bars at 20 µm as indicated.
doi:10.1371/journal.pone.0099410.g008

As shown in the histological stains, there is a paucity of cellular attachment in the control cell-scaffold constructs.

Gene Expression

There was an increase in all mRNA expression levels of chondrogenic markers in both chondrogenic pellets and cell-scaffold constructs tested from week 0 to week 4. It is important to note that there were undetectable levels of expression of collagen type II in the cells prior to plating. In contrast, collagen type II expression was present at week 2 and was increased significantly by week 4 in both pellets and cell-scaffold constructs ($p<0.05$) (Figure 9). However, collagen type I was present at time zero in the cells and by week 4 there was comparable expression levels of collagen type II gene with collagen type I. Collagen type I expression remained consistent over the four-week culture period and any changes across the pellets or cell-scaffold constructs were not statistically significant.

At week 4 cell-scaffold constructs and pellets cultured in control media (i.e. without TGFβ3 and BMP6) had collagen type II gene expression levels that were undetectable, and only low levels of SOX9 and aggrecan genes were expressed. All chondrogenic genes examined were expressed at a higher level when cultured in chondrogenic media. The expression of collagen type II and aggrecan at week 4 were significantly greater in the chondrogenic group compared with the control group in both pellets and cell-scaffold constructs ($p<0.05$). Figure 10 illustrates the chondrogenic gene expression data between of chondrogenic media and control media groups at week 4.

Discussion and Conclusion

This study demonstrates the infrapatellar fat pad is a reliable and abundant source of adipose stem cells with chondrogenic differentiation capacity that can readily be accessed during surgery as an autologous material. The volume of the material produced means that small harvest of autologous IPFP can yield adequate stem cell numbers for the possible repair of quite substantial areas of cartilage damage. Table 1 shows the studies that have used IPFP for chondrogenesis in the past and the combination of TGFβ3 and BMP6 is unique. In a previous study, we have characterized the chondrogenesis of IPFP-ASCs using this combination of growth factors to demonstrate their chondrogenic potential [37]. In this study we have demonstrated the ability for these cells to undergo chondrogenesis not only by themselves in pellet form but also attach, proliferate and differentiate on a 3D printed chitosan scaffold which may serve as a delivery mechanism for these cells into a site of cartilage repair.

In our experiments, all three samples of IPFP-ASCs did not express detectable levels of collagen type II and SOX9 genes at week 0. However, it is clear the expression of these markers increased substantially over four weeks ($p<0.05$). The increase in collagen type II expression was significant. Collagen type II expression remained undetectable at week 4 in the control group. This suggests the addition of TGFβ3 and BMP6 has had a profound effect on chondrogenic differentiation and the stimulation of production of collagen type II in the IPFP-ASCs. The macroscopic changes of the cell aggregates and the morphological changes in the cells also clearly demonstrated the progressive

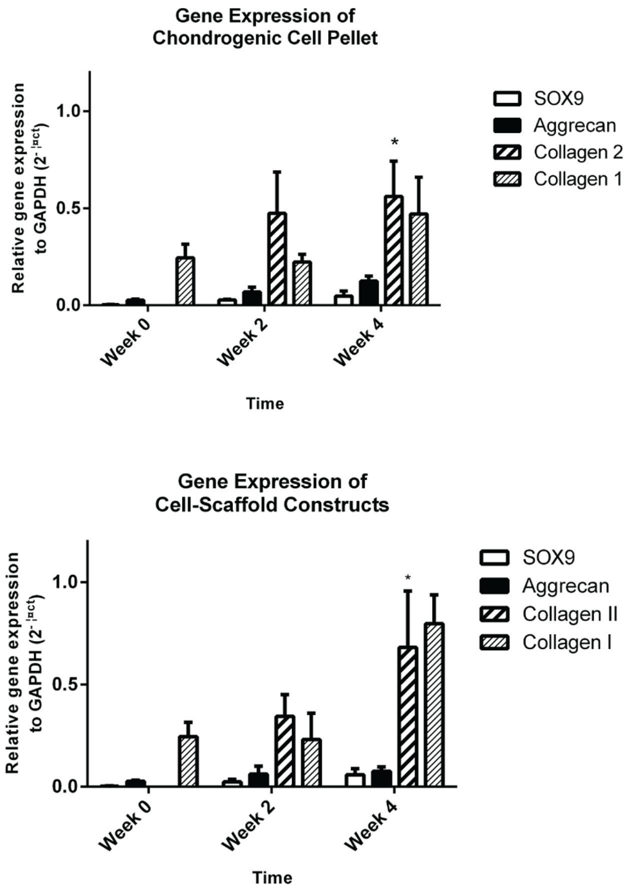

Figure 9. Relative gene expression of chondrogenic pellets and cell-scaffold constructs over time. Collagen type II gene expression, which is not expressed at week 0, is significantly increased over time in both pellet and cell-scaffold cultures (*$p<0.05$; Friedman's test). Other chondrogenic genes such as aggrecan and SOX9 all increase over time however they were less significant. Collagen type I was present from the outset in both cultures and remained elevated throughout, however changes were not statistically significant. These results represent 3 separate experiments using 3 separate biological replicates ($N=3$) as well as triplicate internal replicates for each qPCR reaction. Mean plotted with error bars representing the SEM.
doi:10.1371/journal.pone.0099410.g009

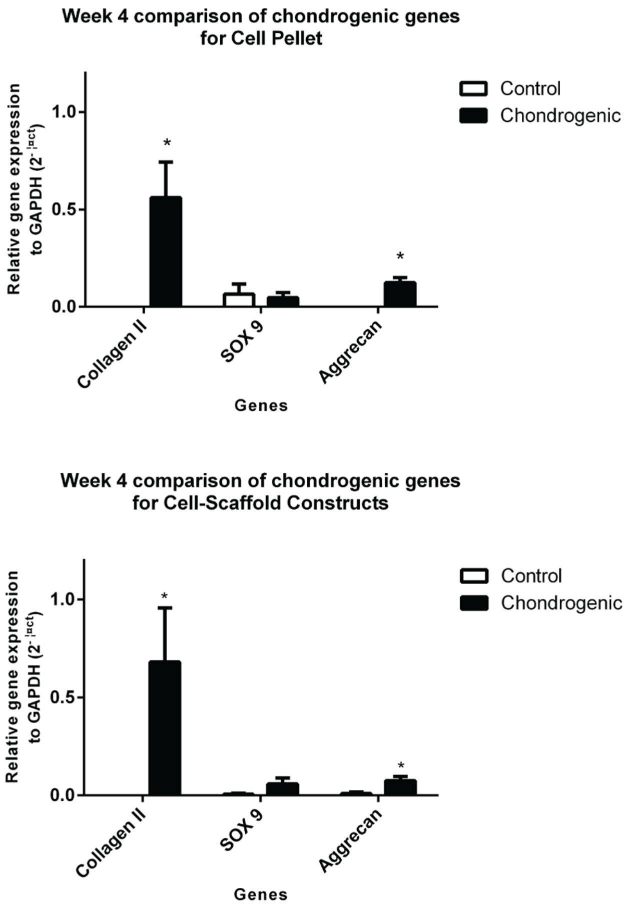

Figure 10. Relative chondrogenic gene expression between control and chondrogenic pellets and cell-scaffold constructs at 4 weeks. Collagen type II gene was not expressed in all control cultures for both pellets and cell-scaffolds at week 4. Both collagen type II and aggrecan genes were significantly increased by week 4 over control groups (*$p<0.05$; t-test). The increase in SOX9 was less significant between the control groups and the chondrogenic groups at 4 weeks. These results represent 3 separate experiments using 3 separate biological replicates ($N=3$) as well as triplicate internal replicates for each qPCR reaction. Mean plotted with error bars representing the SEM.
doi:10.1371/journal.pone.0099410.g010

development of a chondrogenic phenotype. Collagen type I expression remained unchanged from pre-plated cells to week 4 (under chondrogenic conditions). The expression of collagen type I by IPFP-ASCs is consistent with previous studies using these cells [32,38,39,40].

Histologically, there is evidence of some co-localization of collagen type II and type I expression which may provide evidence of early developmental progression at 4 weeks *in vitro*, of the cell-scaffold constructs toward a more chondrogenic phenotype. Collagen type I expression was important to investigate, not only because it is found in fibrocartilage, but because it is expressed in early chondrogenesis as part of the transformation that occurs from mesenchymal cells to chondrocytes. Therefore, collagen type 1 expression is also a marker of early chondrogenesis. This is consistent with the pre-natal development of the knee joint, which starts with a condensation of the mesenchyme between the two long bones prior to the distinct development of the articular surfaces of the long bones [41]. In our study, cell-matrix constructs were maintained for only four weeks and may indicate the need to extend the time period for further clarification of the *in vitro* development sequence. Changes to the composition and structure of the scaffold over time may also impact the production of collagen type I in the cells. This possibility was not investigated in this study, however similar observations were made by other studies of this nature [32].

SOX9 plays a significant role in chondrogenesis, and is also present in other tissues such as the notochord, otic vesicle, neural tube, brain and the developing gonads [42]. In terms of chondrogenesis, it is an important transcription factor in the activation of collagen type II gene during the process of chondrogenesis [42]. Collagen type II is predominantly found in adult articular (hyaline) cartilage and also occurs to a smaller extent in fibrocartilage tissue such as intervertebral disc and meniscus [43]. The presence of SOX 9 in our study is consistent with the chondrogenic differentiation of the IPFP-ASCs under the influence of TGFβ3 and BMP6. The concomitant increase in collagen type II gene expression, and in particular its continued increase over 4 weeks in culture is indicative of the beginning of hyaline cartilage formation. Furthermore the expression of

Table 1. List of studies using IPFP as the stem cell source and the growth factors used.

Study	Cell Source	Culture Type	Growth Factors Used
Dragoo et al (2003) [29]	IPFP-ASC	Micromass with fibrin	IGF1/FGF2
Khan et al (2008) [39]	IPFP-ASC	3D cell aggregate	IGF1/TGFβ3
Lee et al (2008) [38]	IPFP-ASC	Pellet culture	TGFβ1/BMP7
Jurgens et al (2009) [32]	IPFP-ASC	3D PLA-CPL Scaffold	TGFβ1
Buckley et al (2010) [40]	IPFP-ASC (Porcine)	Agarose hydrogel	TGFβ3

Abbreviations: IGF (insulin growth factor), TGFβ (transforming growth factor beta), BMP (bone morphogenetic protein), PLA-CPL (poly L-lactic-co-E-caprolactone).
doi:10.1371/journal.pone.0099410.t001

collagen type II and aggrecan protein in the extracellular matrix, as demonstrated by immunohistochemistry, is a hallmark of hyaline cartilage production [43]. This indicates that the phenotypic expression of the gene products is conclusive for chondrogenesis and hyaline cartilage formation.

The limited the number of biological samples available for this study may attribute to the variability in qPCR results. Ideally, IPFPs from healthy individuals may provide different expression profiles and it has been suggested that mesenchymal stem cells obtained from patients with advanced osteoarthritis reduces its chondrogenic potential [44]. Similar limitations were noted by a previous study in the use of fat pad obtained from patients with osteoarthritis and the potential loss of chondrogenic potential of ASCs [32]. However, it was impossible due to ethical reasons for us to compare the chondrogenic potential of ASCs from healthy versus osteoarthritic IPFP as it involves removing healthy tissue from donors. Therefore, the most available sources of IPFPs were from patients with osteoarthritis undergoing total knee replacements. However, to improve the quality of fat pad, patients with previous surgery to the knee were excluded to reduce the possibility of fibrosis and scar tissue formation within the fat pad. Likewise, patients with autoimmune or inflammatory arthritis undergoing total knee replacements were excluded to reduce the impact of either their disease process or specific auto-immune medications on the potential for cells within the IPFP to proliferate and differentiate. Despite controlling for those factors, the disease process of osteoarthritis may still affect the behavior of cells within the IPFP. If the development of stem cells and biomaterials could improve the management of patients with osteoarthritis, then the use of IPFP from patients with osteoarthritis becomes clinically relevant and applicable.

Although previous studies using IPFP-ASCs have successfully induced chondrogenesis using various growth factors and culture methods, our results differ in the formation of a 'cap' of cartilaginous-like tissue above the chitosan scaffold. The 250 µm porosity of the chitosan scaffold may have limited the penetration of the IPFP-ASCs into the 3D chitosan lattice scaffold due to the self-aggregation nature and extracellular matrix production ability of these cells. These two factors, based on the observations we have seen in this study, influences how the cells behave and attach to the scaffold. In contrast, cells cultured in control media did not exhibit self-aggregation and extracellular matrix production abilities as those under chondrogenic influence, and therefore did not seem to adhere to the chitosan scaffold well, resulting in a paucity of cellular attachment as shown in the histological samples. Therefore, it seems a porosity of 250 µm is more than sufficient for non-chondrogenic cells to penetrate and pass through the scaffold network. However, once cells undergo chondrogenic differentiation, wider pore sizes may be required to accommodate cellular aggregation and enhanced extracellular matrix production to achieve full penetration of the chitosan scaffold, Indeed, in this study, IPFP-ASCs may have not utilized the chitosan as a substitute for the extracellular matrix which they inherently produced, but rather seemed to use the chitosan scaffold for cellular support only. It is possible to capitalize on this interaction such that initially, the 3D nature of the scaffold provides a platform for the cells to naturally aggregate, form a spheroid-like structure and expand in the framework of the scaffold. We have seen this phenomenon in micromass cultures of these cells without scaffold [38,39]. We believe this more accurately mimics the behavior of the formation of an osteochondral unit whereby chondrocytes are fully surrounded in their own matrix supported at their base by subchondral bone which provides strength and support. Whether our cell and chitosan construct behaves like a biphasic 'implant' in vivo remains to be seen. Further in vivo studies are required to assess the behavior of this engineered construct.

In conclusion, infrapatellar fat pad-derived adipose stem cells appear to provide an excellent source of cells with chondrogenic potential. Our results demonstrate the combination of TGFβ3 and BMP6 strongly promotes chondrogenesis with these cells in a 3D chitosan scaffold. This cell-scaffold construct may provide the basis of a viable chondral graft suitable for in vivo implantation.

Author Contributions

Conceived and designed the experiments: KY RF KT SEM GGW PFMC DEM. Performed the experiments: KY RF KT JC. Analyzed the data: KY KT AQ DEM. Contributed reagents/materials/analysis tools: KY KT SEM GGW JC PFMC DEM. Wrote the paper: KY KT PFMC DEM.

References

1. Breinan HA, Martin SD, Hsu HP, Spector M (2000) Healing of canine articular cartilage defects treated with microfracture, a type-II collagen matrix, or cultured autologous chondrocytes. J Orthop Res 18: 781–789.
2. Steadman JR, Rodkey WG, Rodrigo JJ (2001) Microfracture: surgical technique and rehabilitation to treat chondral defects. Clin Orthop Relat Res: S362–369.
3. Steadman JR, Miller BS, Karas SG, Schlegel TF, Briggs KK, et al. (2003) The microfracture technique in the treatment of full-thickness chondral lesions of the knee in National Football League players. J Knee Surg 16: 83–86.
4. Hangody L, Fules P (2003) Autologous osteochondral mosaicplasty for the treatment of full-thickness defects of weight-bearing joints: ten years of experimental and clinical experience. J Bone Joint Surg Am 85-A Suppl 2: 25–32.
5. Brittberg M, Lindahl A, Nilsson A, Ohlsson C, Isaksson O, et al. (1994) Treatment of deep cartilage defects in the knee with autologous chondrocyte transplantation. N Engl J Med 331: 889–895.
6. Zheng MH, Willers C, Kirilak L, Yates P, Xu J, et al. (2007) Matrix-induced autologous chondrocyte implantation (MACI): biological and histological assessment. Tissue Eng 13: 737–746.
7. Bedi A, Feeley BT, Williams RJ III (2010) Management of articular cartilage defects of the knee. J Bone Joint Surg Am 92: 994–1009.

8. Bae DK, Yoon KH, Song SJ (2006) Cartilage healing after microfracture in osteoarthritic knees. Arthroscopy 22: 367–374.
9. Mithoefer K, Williams RJ III, Warren RF, Wickiewicz TL, Marx RG (2006) High-impact athletics after knee articular cartilage repair: a prospective evaluation of the microfracture technique. Am J Sports Med 34: 1413–1418.
10. Mithoefer K, Williams RJ III, Warren RF, Potter HG, Spock CR, et al. (2005) The microfracture technique for the treatment of articular cartilage lesions in the knee. A prospective cohort study. J Bone Joint Surg Am 87: 1911–1920.
11. Gooding CR, Bartlett W, Bentley G, Skinner JA, Carrington R, et al. (2006) A prospective, randomised study comparing two techniques of autologous chondrocyte implantation for osteochondral defects in the knee: Periosteum covered versus type I/III collagen covered. Knee 13: 203–210.
12. Behrens P, Bitter T, Kurz B, Russlies M (2006) Matrix-associated autologous chondrocyte transplantation/implantation (MACT/MACI)-5-year follow-up. Knee 13: 194–202.
13. Zaslav K, Cole B, Brewster R, DeBerardino T, Farr J, et al. (2009) A prospective study of autologous chondrocyte implantation in patients with failed prior treatment for articular cartilage defect of the knee: results of the Study of the Treatment of Articular Repair (STAR) clinical trial. Am J Sports Med 37: 42–55.
14. Bartlett W, Skinner JA, Gooding CR, Carrington RW, Flanagan AM, et al. (2005) Autologous chondrocyte implantation versus matrix-induced autologous chondrocyte implantation for osteochondral defects of the knee: a prospective, randomised study. J Bone Joint Surg Br 87: 640–645.
15. Marcacci M, Berruto M, Brocchetta D, Delcogliano A, Ghinelli D, et al. (2005) Articular cartilage engineering with Hyalograft C: 3-year clinical results. Clin Orthop Relat Res: 96–105.
16. Gobbi A, Kon E, Berruto M, Filardo G, Delcogliano M, et al. (2009) Patellofemoral full-thickness chondral defects treated with second-generation autologous chondrocyte implantation: results at 5 years' follow-up. Am J Sports Med 37: 1083–1092.
17. Chung C, Burdick JA (2008) Engineering cartilage tissue. Adv Drug Deliv Rev 60: 243–262.
18. Goessler UR, Bugert P, Bieback K, Sadick H, Baisch A, et al. (2006) In vitro analysis of differential expression of collagens, integrins, and growth factors in cultured human chondrocytes. Otolaryngol Head Neck Surg 134: 510–515.
19. Goessler UR, Bugert P, Bieback K, Baisch A, Sadick H, et al. (2004) Expression of collagen and fiber-associated proteins in human septal cartilage during in vitro dedifferentiation. Int J Mol Med 14: 1015–1022.
20. Darling EM, Athanasiou KA (2005) Rapid phenotypic changes in passaged articular chondrocyte subpopulations. J Orthop Res 23: 425–432.
21. Lee KH, Song SU, Hwang TS, Yi Y, Oh IS, et al. (2001) Regeneration of hyaline cartilage by cell-mediated gene therapy using transforming growth factor beta 1-producing fibroblasts. Hum Gene Ther 12: 1805–1813.
22. Mandl EW, van der Veen SW, Verhaar JA, van Osch GJ (2002) Serum-free medium supplemented with high-concentration FGF2 for cell expansion culture of human ear chondrocytes promotes redifferentiation capacity. Tissue Eng 8: 573–580.
23. Tuan RS, Boland G, Tuli R (2003) Adult mesenchymal stem cells and cell-based tissue engineering. Arthritis Res Ther 5: 32–45.
24. French MM, Rose S, Canseco J, Athanasiou KA (2004) Chondrogenic differentiation of adult dermal fibroblasts. Ann Biomed Eng 32: 50–56.
25. Asakura A, Komaki M, Rudnicki M (2001) Muscle satellite cells are multipotential stem cells that exhibit myogenic, osteogenic, and adipogenic differentiation. Differentiation 68: 245–253.
26. Nakahara H, Goldberg VM, Caplan AI (1991) Culture-expanded human periosteal-derived cells exhibit osteochondral potential in vivo. J Orthop Res 9: 465–476.
27. Zuk PA, Zhu M, Ashjian P, De Ugarte DA, Huang JI, et al. (2002) Human adipose tissue is a source of multipotent stem cells. Mol Biol Cell 13: 4279–4295.
28. Ogawa R, Mizuno S (2010) Cartilage regeneration using adipose-derived stem cells. Curr Stem Cell Res Ther 5: 129–132.
29. Dragoo JL, Samimi B, Zhu M, Hame SL, Thomas BJ, et al. (2003) Tissue-engineered cartilage and bone using stem cells from human infrapatellar fat pads. J Bone Joint Surg Br 85: 740–747.
30. Awad HA, Wickham MQ, Leddy HA, Gimble JM, Guilak F (2004) Chondrogenic differentiation of adipose-derived adult stem cells in agarose, alginate, and gelatin scaffolds. Biomaterials 25: 3211–3222.
31. Zuk PA, Zhu M, Mizuno H, Huang J, Futrell JW, et al. (2001) Multilineage cells from human adipose tissue: implications for cell-based therapies. Tissue Eng 7: 211–228.
32. Jurgens WJ, van Dijk A, Doulabi BZ, Niessen FB, Ritt MJ, et al. (2009) Freshly isolated stromal cells from the infrapatellar fat pad are suitable for a one-step surgical procedure to regenerate cartilage tissue. Cytotherapy 11: 1052–1064.
33. Suh JK, Matthew HW (2000) Application of chitosan-based polysaccharide biomaterials in cartilage tissue engineering: a review. Biomaterials 21: 2589–2598.
34. Abarrategi A, Lopiz-Morales Y, Ramos V, Civantos A, Lopez-Duran L, et al. (2010) Chitosan scaffolds for osteochondral tissue regeneration. J Biomed Mater Res A 95: 1132–1141.
35. Woodfield TB, Malda J, de Wijn J, Peters F, Riesle J, et al. (2004) Design of porous scaffolds for cartilage tissue engineering using a three-dimensional fiber-deposition technique. Biomaterials 25: 4149–4161.
36. Estes BT, Diekman BO, Gimble JM, Guilak F (2010) Isolation of adipose-derived stem cells and their induction to a chondrogenic phenotype. Nat Protoc 5: 1294–1311.
37. Felimban R, Ye K, Traianedes K, Di Bella C, Crook J, et al. (2014) Differentiation of stem cells from human infrapatellar fat pad: Characterization of cells undergoing chondrogenesis. Tissue Eng Part A.
38. Lee SY, Nakagawa T, Reddi AH (2008) Induction of chondrogenesis and expression of superficial zone protein (SZP)/lubricin by mesenchymal progenitors in the infrapatellar fat pad of the knee joint treated with TGF-beta1 and BMP-7. Biochem Biophys Res Commun 376: 148–153.
39. Khan WS, Tew SR, Adesida AB, Hardingham TE (2008) Human infrapatellar fat pad-derived stem cells express the pericyte marker 3G5 and show enhanced chondrogenesis after expansion in fibroblast growth factor-2. Arthritis Res Ther 10: R74.
40. Buckley CT, Vinardell T, Thorpe SD, Haugh MG, Jones E, et al. (2010) Functional properties of cartilaginous tissues engineered from infrapatellar fat pad-derived mesenchymal stem cells. J Biomech 43: 920–926.
41. Merida-Velasco JA, Sanchez-Montesinos I, Espin-Ferra J, Rodriguez-Vazquez JF, Merida-Velasco JR, et al. (1997) Development of the human knee joint. Anat Rec 248: 269–278.
42. Lefebvre V, de Crombrugghe B (1998) Toward understanding SOX9 function in chondrocyte differentiation. Matrix Biol 16: 529–540.
43. Eyre D (2002) Collagen of articular cartilage. Arthritis Res 4: 30–35.
44. Murphy JM, Dixon K, Beck S, Fabian D, Feldman A, et al. (2002) Reduced chondrogenic and adipogenic activity of mesenchymal stem cells from patients with advanced osteoarthritis. Arthritis Rheum 46: 704–713.
